# What Can Deep Neural Networks Teach Us About Embodied Bounded Rationality

**DOI:** 10.3389/fpsyg.2022.761808

**Published:** 2022-04-25

**Authors:** Edward A. Lee

**Affiliations:** Electrical Engineering and Computer Sciences, University of California, Berkeley, Berkeley, CA, United States

**Keywords:** bounded rationality, embodied cognition, neural networks, artificial intelligence, computation

## Abstract

“Rationality” in Simon's “bounded rationality” is the principle that humans make decisions on the basis of step-by-step (algorithmic) reasoning using systematic rules of logic to maximize utility. “Bounded rationality” is the observation that the ability of a human brain to handle algorithmic complexity and large quantities of data is limited. Bounded rationality, in other words, treats a decision maker as a machine carrying out computations with limited resources. Under the principle of embodied cognition, a cognitive mind is an *interactive* machine. Turing-Church computations are not interactive, and interactive machines can accomplish things that no Turing-Church computation can accomplish. Hence, if “rationality” is computation, and “bounded rationality” is computation with limited complexity, then “embodied bounded rationality” is both more limited than computation and more powerful. By embracing interaction, embodied bounded rationality can accomplish things that Turing-Church computation alone cannot. Deep neural networks, which have led to a revolution in artificial intelligence, are both interactive and not fundamentally algorithmic. Hence, their ability to mimic some cognitive capabilities far better than prior algorithmic techniques based on symbol manipulation provides empirical evidence for the principle of embodied bounded rationality.

## 1. Introduction

From a computer science perspective, a rational process is step-by-step reasoning using clearly explicable rules of logic. Intractability arises when the number of steps or the amount of data that has to be stored gets too large. Simon's bounded rationality (Simon, 1972) can be interpreted as a recognition of the difficulty that the human mind has in carrying out such rational processes.

Computers, on the other hand, are superbly matched to this sort of rational process. When a decision problem can be formulated as an optimization problem with a clearly defined cost function, an algorithm can often be devised to make an optimal decision. These algorithms are rational in the same sense; they are step-by-step procedures where each step is justified using explicable rules of logic. Such algorithms have repeatedly proven tractable to computers even when hopelessly intractable to humans. When they prove intractable to computers as well, we can often refine them with heuristics and approximations that lead to close to optimal solutions, but even these heuristics are explicable and hence rational.

Among the successes of such computer-driven decision-making are those that lie in the field of optimal control, where a machine makes decisions in response to sensor inputs, and these decisions are used to drive actuators that change the physical world in such a way as to feed back into the sensors. Self-driving cars, industrial robots, automated trains, and the electric power grid are all examples of such systems. The algorithmic decision-making in these systems interacts with the physical world in such a tight feedback loop that the behavior of the computer cannot be decoupled from the behavior of its physical environment. A whole branch of engineering called “cyber-physical systems” (CPS) has arisen to address the technological problems around such embodied robots (Lee, 2008).

Today, to the surprise and, in some cases, extreme frustration of many researchers, many optimization solutions in engineering are being routinely outperformed by deep neural networks (DNNs). While DNNs are often described as “algorithms,” they do not rise to the level of “rational decision making” in the same sense. Although DNNs are typically realized algorithmically on computers, when a DNN produces a result, e.g., classifying an image as a Stop sign, there is no sequence of logical steps that you can point to that rationally leads to the classification. The classification is more like intuition than rationality.

The AlphaGo project (Silver et al., 2016) conclusively demonstrated this principle. The game of Go is notoriously intractable as an optimization problem, and heuristics lead to amateurish play. The best players do not arrive at their moves using a sequence of logical steps, and neither does AlphaGo. More precisely, although AlphaGo is realized as a computer program, that program does not describe any rational decision-making that has anything at all to with the game of Go. Instead, it describes how to build and use a very large data set, refining it by having computers play millions of games of Go with each other. The ability of the program to beat the best Go Masters lies much more in the learning process and the resulting data than in the sequence of rational steps that make and make use of the data. The data has been cultivated in much the same way that a Go Master builds expertise, by practicing.

The centrality of data here is easily and frequently misunderstood. The mantra that “data is the new oil” suggests that data is a resource lying all around us waiting to be exploited. Suppose, for example, that we had stored on disk drives somewhere a record of all the Go games ever played by Go Masters. This would certainly be valuable, but the AlphaGo team did not have such a data set, and their result would likely have been less spectacular had they trained their DNNs on that data set. If they had such a data set, they could have trained a DNN easily because each move in each board position is clearly labeled as a “winning” or “losing” move by the final outcome of the game. But that is not what they did. Instead, they programed their machines to play against each other. The first few million games were amateurish, but through the magic of backpropagation, each game refined the data driving the decisions such that each game got better. The data was not mined, it was created.

The process of training the AlphaGo machines is interactive, not observational. It is first person, not third person. By analogy, a human will never acquire the ability to outperform Go Masters by just watching masters play Go. The human has to interact with Go Masters to become a Go Master. Interaction is more powerful than observation. Not only do humans learn better by doing, so do machines.

The principle of embodied cognition puts interaction front-and-center. The mind is not a process in a brain observing the world through sensors. Instead, the mind is an interaction between processes in a brain and the world around it (Thelen, 2000). The kinds of problems that DNNs excel at are precisely those where interaction is front-and-center. And the decisions made by DNNs are frustratingly inexplicable, resisting any label as rational decisions.

In this article, I will show from several perspectives that interaction is more powerful than observation. There are things that can be accomplished through interaction that are impossible through observation. I will give technical and mathematical examples that are not possible without interaction.

I will also show that interaction can occur without algorithms. Although DNNs can be realized by computers, these realizations are brute-force simulations of processes that are not fundamentally algorithmic. The field of reservoir computing (Tanaka et al., 2019), for example, offers very different architectures that have little resemblance to Turing-Church computations and would be hard to describe as rational decision makers in the sense considered here. The field of feedback control, which is fundamentally about interaction, does not fundamentally need computers nor algorithms. Indeed, its earliest applications in the 1920s through the 1950s predate digital computers.

Proponents of embodied cognition often use the term “computation” much more broadly than I am using it here to mean any sort of information processing (Dodig-Crnkovic and Giovagnoli, 2013; Müller and Hoffmann, 2017; Dodig-Crnkovic, 2018). Any dynamic process that reacts to sensed information about its environment is capable of such “morphological computing” (Pfeifer and Bongard, 2007) or “natural computing” (Müller and Hoffmann, 2017). Such computation is performed by every living organism (Maturana and Varela, 1980; Stewart, 1995) and many non-living organisms (a thermostat, for example), and hence is much too broad to bear much if any relationship to bounded rationality in the sense of Simon (1972). In this article, “computation” will be limited to the meaning given by Turing and Church, as done for example by Piccinini (2007), and I will show in Section 4 that this meaning is not the same as information processing. I will argue that the Turing-Church meaning of “computation” does not even include many of the processes we accomplish today using digital computers. But it is this sense that matches the bounded rationality of Simon.

In the prevailing philosophy of science, observation trumps interaction. We are taught that the best science is objective, not subjective. Let the data speak for itself. Design your instruments to minimally disrupt what you are observing. But science also teaches us that observation without interaction is impossible. My claim is that it is also undesirable. We can accomplish much more if we embrace feedback and interaction.

The main contributions of this article are to point out that Turing-Church computations are objective, observational, and non-interactive processes; to clarify that an algorithm is the specifications of what a Turing-Church computation does; to show in several ways that first-person interaction, i.e., a feedback system, can accomplish things that no Turing-Church computation can; to argue that deep neural networks are feedback systems and are not fundamentally algorithmic; and to argue that the efficacy of DNNs on certain cognitive tasks provides empirical support for the thesis of embodied bounded rationality.

## 2. Bounded Rationality

In the 1970s, Herbert Simon challenged the prevailing dogma in economics, which assumed that agents act rationally. His key insight, for which he got the Nobel Prize in economics, was that those agents (individuals and organizations) do not have the capability to make the kinds of rational decisions that economists assumed they would. In his words:

Theories that incorporate constraints on the information-processing capacities of the actor may be called theories of bounded rationality (Simon, 1972).

He identified three limitations: uncertainty about the consequences that would follow from alternative decisions, incomplete information about the set of alternatives, and complexity preventing the necessary computations from being carried out. He argued that “these three categories tend to merge,” using the game of chess as an example and saying that the first and second, like the third, are fundamentally an inability to carry out computation with more than very limited complexity:

What we refer to as “uncertainty” in chess or theorem proving, therefore, is uncertainty introduced into a perfectly certain environment by inability—computational inability—to ascertain the structure of that environment (Simon, 1972).

Three decades later, he reaffirmed this focus on the process of reasoning:

When rationality is associated with reasoning processes, and not just with its products, limits on the abilities of Homo sapiens to reason cannot be ignored (Simon, 2000).

Reasoning and rationality as computation are central to his theory, and he argued that economists' assumptions that agents would maximize expected utility was unrealistic in part because that maximization is intractable to a human mind.

## 3. Algorithms and Computation

What is an algorithm? Merriam-Webster gives this definition: “a step-by-step procedure for solving a problem or accomplishing some end.” Despite the simplicity of this definition, the term is widely used more broadly. Domingos (2015), for example, in his book *The Master Algorithm*, states that Newton's second law is an algorithm. Often expressed as *F* = *ma*, force equals mass times acceleration, Newton's second law is *not* an algorithm. There are no steps, there is no procedure, and there is no end. Instead, Newton's second law is a relation between two continuously varying quantities, force and acceleration, where the latter quantity expresses a rate of change of velocity, which in turn expresses a rate of change of position. Domingos seems to use the word “algorithm” to mean anything that is formally expressible. In this article, I will use the term “algorithm” in a narrower manner consistent with the Merriam-Webster definition.

Newton's second law is a differential equation. Acceleration is the second derivative of position. Not only is a differential equation not an algorithm, but many differential equations express behaviors for which *there is no algorithm*. Every algorithm that attempts to *simulate* a process described by such a differential equation is flawed. Newton's second law is a linear differential equation for which, for many input force functions, we can find a closed-form solution. Once we have such a solution, we can devise an algorithm that gives the position at any chosen point in time. However, for non-trivial force inputs, and for most non-linear differential equations, there is no such closed-form solution, and every algorithmic approximation exhibits arbitrarily large errors. Non-linear differential equations, in particular, often exhibit chaotic behavior, where arbitrarily small errors at any step become arbitrarily large errors in future steps. The discovery of such chaotic behavior is attributed to Lorenz (1963), who was frustrated by the inability of computer models to predict weather more than a few days in advance. The differential equations modeling the thermodynamics of weather are chaotic, and every algorithmic approximation develops arbitrarily large errors over time.

While time is central to differential equations, it is irrelevant to algorithms. The steps of an algorithm are discrete, entirely separable from one another, and the time it takes to complete a step is irrelevant to whether the algorithm is being correctly carried out. In contrast, in an interactive system or a feedback system where part of the interaction is a physical process, time plays a major role. Hence, under the principle of embodied cognition, time is central to cognition, a point forcefully made by Esther Thelen:

It is precisely the continuity in time of the embedded and coupled dynamic systems essential for fluid, adaptive behavior that gives meaning to the notion of an embodied cognition (Thelen, 2000).

What is the relationship between algorithms and computation? Here again, I will stick to a rigorous use of this term, adopting the meaning established by Turing (1936) and Church (1932). In this meaning, a computation is a step-by-step procedure (i.e., a carrying-out of an algorithm) operating on digital information that terminates and gives an answer. What is now called the Church-Turing thesis states that every such computation can be computed by a Turing machine, a machine that realizes the algorithm. Turing showed that there is a particular Turing machine, or, equivalently, a particular algorithm, that can realize any other Turing machine. This machine is called a “universal Turing machine.” Given enough time and memory, any modern computer can realize a universal Turing machine.

Unfortunately, many people misrepresent the universal Turing machine, calling it simply a “universal machine,” and stating that it can realize any other machine. For example, in his book *Tools for Thought*, Howard Rheingold states,

The digital computer is based on a theoretical discovery known as “the universal machine,” which is not actually a tangible device but a mathematical description of a machine capable of simulating the actions of any other machine (Rheingold, 2000, p. 15).

Rheingold misleads by speaking too broadly about machines. There is no universal machine, mathematical or otherwise. A universal Turing machine can only perform computations.^1^

With regard to computation, humans are much more limited than computers. Computers have no difficulty taking billions of steps in an algorithm to solve a problem, whereas humans struggle with a few dozen. Algorithmic reasoning may seem like the epitome of thought, but if it is, then humans fall far short of that epitome. So far short, in fact, that Simon may have not gotten it quite right. If human decisions are the result of a limited amount of computation, then it is an extremely limited amount. What if they are not the result of computation at all?

Kahneman, in his book, *Thinking Fast and Slow*, identifies two distinct human styles of thinking, a fast style (System 1) and a slow style (System 2). The slow style is capable of algorithmic reasoning, but the fast style, which is more intuitive, is responsible for many of the decisions humans make. It turns out that many of today's artificial intelligences (AIs) more closely resemble System 1 than System 2. Even though they are realized on computers, they do not reach decisions by algorithmic reasoning.

## 4. Information Processing Is Not (Necessarily) Computation

“Computation,” in the sense that I am using the term in this article, is not the same as information processing, in the sense used in Dodig-Crnkovic and Giovagnoli (2013), Müller and Hoffmann (2017), and Dodig-Crnkovic (2018). In this article, computation is (a) algorithmic (consisting of a sequence of discrete steps, where each step is drawn from a finite set of possible operations); (b) terminating; (c) operating on discrete data (the inputs, outputs, and intermediate states are all drawn from countable sets); and (d) non-interactive (inputs are available at the start and outputs at termination). Turing-Church computation has all four of these properties. Under this definition, the set of all possible computations is countable. The core results in the theory of computation (e.g., undecidability, complexity measures, and the universality of Turing machines) all depend on this countability.

In Lee (2017) (Chapter 7), I define “information” as “resolution of alternatives.” Using Shannon information theory, I point out that information need not be discrete. The alternatives may lie in a finite, countable, or uncountable set. I show that measurements of information (entropy) are incomparable when the alternatives lie in a finite or countable set vs. when they lie in an uncountable set. There is an infinite offset between these two measures of information. In particular, if the set of alternatives is countable, then entropy gives the expected number of bits needed to encode a selected alternative. This number of bits is a measure of the amount of information gained by observing a selected alternative. However, if the set of alternatives is uncountable, then entropy can still be finite, but it no longer represents a number of bits needed to encode a selected alternative. In fact, an infinite number of bits is required. Nevertheless, this entropy can still be interpreted as a measure of the amount of information in an observation of an alternative, and these amounts can be compared with each other, but these amounts are always infinitely larger than the amount of information in an observation drawn from a countable set of alternatives.

Many mistakes are made in the literature by ignoring this infinite offset. For example, Lloyd (2006) says about the second law of thermodynamics, “It states that each physical system contains a certain number of bits of information—both invisible information (or entropy) and visible information—and that the physical dynamics that process and transform that information never decrease that total number of bits.” But the second law works absolutely unmodified if the underlying random processes are continuous, in which case the set of alternatives is uncountable, and the information is not representable in bits. The same mistake is made by Goyal (2012), who states “The fact that [the entropy of a black hole] is actually finite suggests that the degrees of freedom are not non-denumerably infinite.” But the entropy of a black hole given by Bekenstein (1973) is based on a continuous probability density, so its finiteness does not imply countable degrees of freedom. Goyal (2012) continues, stating for example that in quantum physics, “the number of possible outcomes of a measurement *may* be finite or countably infinite” (emphasis added), and then implying that it is *always* finite or countably infinite. Goyal (2012) goes on to assert that “this stands in contrast with the classical assumption that all physical quantities (such as the position of a particle) can take a continuum of possible values.” There are some physical measurements that have only a finite or countable number of outcomes, such as the spin of an electron, but position of a particle is not one of them. The Schrödinger equation operates in a time and space continuum and the wave function describing position is reasonably interpreted as a probability *density* function governing an uncountable number of possible alternatives. The discreteness of time and space is a later overlay on quantum theory that remains controversial and is not experimentally supported. Goyal takes a leap of faith, concluding “hence, discreteness challenges the classical idea that the continua of space and time are the fundamental bedrock of physical reality.” In contrast, Dodig Crnkovic (2012) observes that “information is both discrete and continuous.”

Information that lies in a continuum of alternatives can be operated on by processes that are neither algorithmic nor terminating. Ordinary differential equation models of the physical world can be interpreted as performing such operations. Such information processing is not, however, computation. Chaos theory shows that such information processing cannot even be approximated with bounded error by computation (Lee, 2017, Chapter 10).

Given these facts, we are forced to make one of two choices: either (A) information processing is richer than computation, or (B) the physical world does not have uncountable alternatives, Hypothesis (B) is sometimes called “digital physics” (Lee, 2017, Chapter 8). Some physicists and computer scientists go further and claim that everything in the material world *is actually* a Turing-Church computation.^2^ I have previously shown, however, that hypothesis (B) is not testable by experiment unless it is *a priori* true (Lee, 2017, Chapter 8). Specifically, the Shannon channel capacity theorem tells us that every noisy measurement conveys only a finite number of bits of information, and therefore can only distinguish elements from a countable set of alternatives. Hence, hypothesis (B) is scientific, in the sense of Popper (1959), only if it is *a priori* true. Hence, hypothesis (B) is a matter of faith, not science.

If anything in the physical world forms a continuum (time or space, for example), then noise in measurements remains possible, no matter how good the measurement apparatus becomes. This follows from the incompleteness of determinism (Lee, 2016). A noiseless measurement of some physical system would have to be deterministic, in the sense that the same physical state should always yield the same measurement result. However, I have shown in Lee (2016) that any set of deterministic models of the physical world that includes both discrete and continuous alternatives and that is rich enough to include Newton's laws is incomplete. It does not contain its own limit points. Non-determinism, therefore, is inescapable unless digital physics is *a priori* true and there are no continuous alternatives. This means that at least some measurements will always be vulnerable to noise unless the hypothesis to be tested experimentally is already true. Hence, hypothesis (B) can only be defended by a circular argument.

Hypothesis (B) is not only a matter of faith, but it also a poor choice under the principle of Occam's razor. As I point out in Lee (2020) (Chapter 8), models based only on countable sets may be far more complex than models based on continuums. Diophantine equations, for example, which are widely used in physics, for example to describe the motions of bodies in gravitational fields, are chaotic and exhibit weird gaps when defined over countable sets. A more defensible position, therefore, is hypothesis (A), which allows for information processing as a reasonable model of the physical world without insisting that information processing have the form of computation. This position is also supported by Piccinini (2020) who states, “information processing may or may not be done by computing” (Chapter 6).^3^

## 5. Deep Neural Networks

Deep neural nets (DNNs), which have transformed technology by enabling image classification, speech recognition, and machine translation, to name a few examples, are inspired by the tangle of billions of neurons in the brain and rely on the aggregate effect of large numbers of simple operations. They are, today, mostly realized by computers, and hence are composed of “algorithms” and “computation.” However, to view these realizations as Turing-Church computations is to ignore the role of feedback, a property absent in the Turing-Church model. This role is not incidental. Moreover, it also ignores the possibility that today's realizations of neural networks are brute force computational approximations of information processing that is not, at its root, computational.

A frustrating result of the recent successes in deep neural nets is that people have been unable to provide explanations for many of the decisions that these systems make (Lee, 2020, Chapter 6). In May 2018 a new European Union regulation called the General Data Protection Regulation (GDPR) went into effect with a controversial provision that provides a right “to obtain an explanation of the decision reached” when a decision is solely based on automated processing. Legal scholars, however, argue that this regulation is neither valid nor enforceable (Wachter et al., 2017). In fact, it may not even be desirable. I conjecture that sometime in the near future, someone will figure out how to train a DNN to provide a convincing explanation for *any* decision. This could start with a generative-adversarial network (GAN) that learns to provide explanations that appear to be generated by humans.

Humans are very good at providing explanations for our decisions. But our explanations are often wrong or at least incomplete. They are likely to be *post hoc* rationalizations, offering as explanations factors that do not or cannot account for the decisions we make. This fact about humans is well-explained by Kahneman, whose work on “prospect theory,” like Simon's bounded rationality, challenged utility theory. In prospect theory, decisions are driven more by gains and losses rather than the value of the outcome. Humans, in other words, will make irrational decisions that deliver less value to them in the end. In *Thinking Fast and Slow*, Kahneman offers a wealth of evidence that our decisions are biased by all sorts of factors that have nothing to do with rationality and do not appear in any explanation of the decision.

Kahneman reports, for example, a study of the decisions of parole judges in Israel by Danziger et al. (2011). The study found that these judges, on average, granted about 65 percent of parole requests when they were reviewing the case right after a food break, and that their grant rate dropped steadily to near zero during the time until the next break. The grant rate would then abruptly rise to 65 percent again after the break. In Kahneman's words,

The authors carefully checked many alternative explanations. The best possible account of the data provides bad news: tired and hungry judges tend to fall back on the easier default position of denying requests for parole. Both fatigue and hunger probably play a role (Kahneman, 2011).

And yet, I'm sure that every one of these judges would have no difficulty coming up with a plausible explanation for their decision for each case. That explanation would not include any reference to the time since the last break.

Taleb, in his book *The Black Swan*, cites the propensity that humans have, after some event has occurred, to “concoct explanations for its occurrence after the fact, making it explainable and predictable” (Taleb, 2010). For example, the news media always seems to have some explanation for movements in the stock market, sometimes using the same explanation for both a rise and a fall in prices.

Taleb reports on psychology experiments where subjects are asked to choose among twelve pairs of nylon stockings the one they like best. After they had made their choice, the researchers asked them for reasons for their choices. Typical reasons included color, texture, and feel, but in fact, all twelve pairs were identical.

Taleb also reports on some rather dramatic experiments performed with split-brain patients, those who have undergone surgery where the corpus callosum connecting the two hemispheres of the brain has been severed. Such surgery has been performed on a number of victims of severe epilepsy that have not responded to less aggressive treatments. These experiments support the hypothesis that the propensity for *post hoc* explanations has deep biological roots. An image presented to the left half of the visual field will go to the right side of the brain, and an image presented to the right half of the visual field will go to the left side of the brain. In most people, language is centered in the left half of the brain, so the patient will only be able to verbalize the right field experience. For example, a patient with a split brain is shown a picture of a chicken foot on the right side and a snowy field on the left side and asked to choose the best association with the pictures. The patient would correctly choose a chicken to associate with the chicken foot and a shovel to associate with the snow. When asked why the patient chose the shovel, the patient would reply that was “for cleaning out the chicken coop.” Taleb concludes,

Our minds are wonderful explanation machines, capable of making sense out of almost anything, capable of mounting explanations for all manner of phenomena, and generally incapable of accepting the idea of unpredictability (Taleb, 2010).

Demanding explanations from AIs could yield convincing explanations for anything, leading us to trust their decisions too much. Explanations for the inexplicable, no matter how plausible, are simply misleading.

Given that humans have written the computer programs that realize the AIs, and humans have designed the computers that execute these programs, why is it that the behavior of the programs proves inexplicable? The reason is that what the programs do is not well-described as algorithmic reasoning, in the same sense that an outbreak of war is not well-described by the interactions of protons and electrons. Explaining the implementation does not explain the decision.

Before the explosive renaissance of AI during the past two decades, AI was dominated by attempts to encode algorithmic reasoning directly through symbolic processing. What is now called “good old-fashioned AI” (GOFAI) encodes knowledge as production rules, if-then-else statements representing the logical steps in algorithmic reasoning (Haugeland, 1985). GOFAI led to the creation of so-called “expert systems,” which were sharply criticized by Dreyfus and Dreyfus (1986) in their book, *Mind Over Machine*. They pointed out, quite simply, that following explicit rules is what novices do, not what experts do. Dreyfus and Dreyfus called the AI practitioners of the time,

false prophets blinded by Socratic assumptions and personal ambition—while Euthyphro, the expert on piety, who kept giving Socrates examples instead of rules, turns out to have been a true prophet after all (Dreyfus and Dreyfus, 1984).

Here, Dreyfus and Dreyfus are reacting (rather strongly) to what really was excessive hyperbole about AI at the time. They were just the tip of a broad backlash against AI that came to be called the “AI winter,” where funding for research and commercial AI vanished nearly overnight and did not recover until around 2010.

DNNs work primarily from examples, “training data,” rather than rules. The explosion of data that became available as everything went online catalyzed the resurgence of statistical and optimization techniques that had been originally developed in the 1960s through 1980s but lay dormant through the AI winter before exploding onto the scene around 2010.

DNNs particularly excel at functions that, in humans, we call perception, for example the ability to classify objects in an image. Stewart (1995) attributes to the Chilean biologist and philosopher Maturana the perspective that, “perception should not be viewed as a grasping of an external reality, but rather as the specification of one.” Indeed, the supervised training process that for a DNN such as Inception, which is distributed by Google as part of their open-source TensorFlow machine learning toolkit, results in a network that *specifies* a taxonomy rather than recognizing an objectively existing one. Because training images are labeled by humans, the resulting taxonomy is familiar to humans.

The techniques behind the AI renaissance are nothing like the production rules of GOFAI. A central one of these techniques, now called backpropagation, first showed up in automatic control problems quite some time ago. Kelley (1960) describes a controller that would carry a spacecraft from Earth's orbit to Mars's orbit around the sun using a solar sail. His controller, a feedback system, bears a striking resemblance to backpropagation, although his formulation is more continuous than the discrete form used in machine learning today. His formulation did not require a digital computer to realize it, and in fact, any computer realization would have been an approximation of his specification.

Based in part on Kelley's work, Bryson et al. (1961) describe a feedback system to control a spacecraft that is re-entering the earth's atmosphere to minimize heating due to friction. They adapted Kelley's method into a multistage technique that closely resembles the backpropagation technique used for DNNs today. The Kelley-Bryson technique was restated in a form closer to its usage today by Dreyfus (1962).

Backpropagation can be thought of as a technique for a system to continuously redesign itself by probing its environment (including its own embodiment) and adapting itself based on the reaction. DNNs realized in software are better thought of as programs that continuously rewrite themselves during their training phase. Today, it is common to freeze the program after the training phase, or to update it only rarely, but this practice is not likely to persist for many applications. Continuing to learn proves quite valuable.

There have been attempts to use machine learning techniques to learn *algorithmic* reasoning, where the result of the training phase is a set of explicable production rules, but these have proven to underperform neural networks. Wilson et al. (2018) created a program that could write programs to play old Atari video games credibly well. Their program generated random mutations of production rules, and then simulated natural selection. Their technique was based on earlier work that evolved programs to develop certain image processing functions (Miller and Thomson, 2000). The Atari game-playing programs that emerge, however, are far less effective than programs based on DNNs. Wilson et al. (2018) admit this, saying that the main advantage of their technique is that the resulting programs are more explainable. The learned production rules provide the explanations.

In contrast, once a DNN has been trained, even a deep understanding of the computer programs that make its decisions does not help in providing an explanation for those decisions. Exactly the same program, with slightly different training, would yield different decisions. So the explanation for the decisions must be in the data that results from the training. But those data take of the form of millions of numbers that have been iteratively refined by backpropagation, a feedback system. The numbers bear no resemblance to the training data and have no simple mapping onto symbols representing inputs and possible decisions. Even a deep understanding of backpropagation does little to explain how the particular set of numbers came about and why they lead to the decisions that they do. Fundamentally, the decisions are not a consequence of algorithmic reasoning.

Today, implementations of DNNs are rather brute force, using enormous amounts of energy and requiring large data centers with a great deal of hardware. The energy consumption of a human brain, in contrast, is quite modest. In an attempt to come closer, there is a great deal of innovation on hardware for machine learning. Some of this hardware bears little resemblance to modern computers and has no discernible roots in Turing-Church computation, using for example analog circuits. Reservoir computing (Tanaka et al., 2019) is a rather extreme example, where a fixed, non-linear system called a reservoir is used as a key part of a neural network. The reservoir can be a fixed physical system, such as a random bundle of carbon nanotubes and polymers. These innovations demonstrate that DNNs are not, fundamentally, Turing-Church computations, and they may eventually be realized by machines that do not resemble today's computers.

Simon developed his theory of bounded rationality well before DNNs, at a time when AI was all about symbolic processing. Newell and Simon (1976) say, “symbols lie at the root of intelligent action, which is, of course, the primary topic of artificial intelligence.” They add, “a physical symbol system has the necessary and sufficient means for general intelligent action.” They go further and commit to the universal machine hypothesis:

A physical symbol system is an instance of a universal machine. Thus the symbol system hypothesis implies that intelligence will be realized by a universal computer (Newell and Simon, 1976).

We now know that this hypothesis is false. DNNs outperform symbolic processing on many problems, particularly on more cognitively difficult problems. Although their realizations in computers arguably use symbols (0 to represent “false” and 1 to represent “true,” for example), those symbols have no relationship to the problem they are solving.

## 6. Interaction and Feedback

In the thesis of embodied cognition, the mind “simply does not exist as something decoupled from the body and the environment in which it resides” (Thelen, 2000). The mind is not a computation that accepts inputs from the environment and produces output, but rather the mind *is* an interaction of a brain with its body and environment. A cognitive being is not an observer, but rather a collection of feedback loops that include the body and its environment. Fundamentally, under this thesis, a cognitive mind is an interactive system.

If “rationality” is computation, and “bounded rationality” is computation with limited resources, then “embodied bounded rationality” is both more limited than computation and more powerful. By embracing interaction, embodied bounded rationality can accomplish things that bounded rationality or even unbounded rationality alone cannot.

Turing-Church computation is not interactive. There is no part of the theory that includes effects that outputs from the computation may have on inputs to the computation. Central to what a computation is, in this theory, is that the inputs are fully available at the start, and that the outputs are available when the computation terminates. If the computation does not terminate, there is no output and the process is not a computation. There is nothing in the formalism that enables the machine to produce intermediate outputs, allow the environment to react and provide new inputs, and then continue by reacting to those new inputs. The “universal” Turing machine proves to be far from universal because it does not include any such interactive machines.

To understand this point, it is critical to realize that the behavior that emerges from an interactive machine is not just a consequence of what the machine does, but also of what the machine's environment does. Hence, the only way to make Turing-Church computations truly “universal,” including interactive machines, is to ensure that their environment is part of the Turing-Church computation. To do this in general, you have to assume digital physics, something you can only do on faith.

The biggest breakthroughs in AI replace the prior open-loop good old-fashioned AI (GOFAI) techniques with interaction and feedback. Here, I use the term “feedback” for interaction where one part of the system provides a stimulus to another part, measures its response, and adjusts its actions to make future responses more closely resemble its goals. Deep neural networks are, fundamentally, feedback systems in this sense, and they yield results of such complexity as to be inexplicable (Lee, 2020, Chapter 6). The algorithms by which they are realized on computers are simply not good descriptions of what they do.

In this section, I will go through a series of illustrations of what can be accomplished with interaction and feedback that is not possible with Turing-Church computation alone. Some of these are quite technical and serve as proofs of the limitations of computation, while others are just better explanations of what is really going on.

### 6.1. Driving a Car

Wegner (1998) gives a simple example that illustrates the limitations of non-interactive machines, driving a car. Consider a cruise control system, which maintains the speed of a car close to a specified setpoint. In an interactive solution, the inputs to this system are measurements of the speed of the car, and the system simply accelerates (opens the throttle) if the speed is too low and decelerates if the speed is too high. The system continuously watches the effects of its actions and continuously corrects by adjusting the throttle. The system automatically compensates for changes in the environment, such as climbing a hill. This is a tight feedback loop, an embodied solution where the “smarts” of the cruise control is in its *interaction* with its body (the car) and its environment (the roadway). This solution is extremely simple, a feedback control system realizable with technology that was patented back in the 1930s (Black, 1934), well before digital computers.

Now, consider solving this problem as a Turing-Church computation without interaction. First, in order to terminate, the problem will only be able to be solved for a finite time horizon, and, to be algorithmic, time will need to be discretized. Assume a car is driven for no more than 2 h on each trip, and that we will get sufficient accuracy if we calculate the throttle level that needs to be applied each 100 ms. The output, therefore, will be a trace of 72,000 throttle levels to apply. What is the input? First, we need as input the elevation gains and losses along the trajectory to be taken by the car during all segments where the cruise control is to be active. We will also need a detailed model of the dynamics of the car, including its weight, the weight of each of the passengers and the contents in the trunk, and how the car responds to opening and closing the throttle. The computation will now need to solve complex differential equations governing the dynamics to calculate what throttle to apply to the car as it moves over the specified trajectory. The simple problem has become a nightmare of complexity requiring a great deal of prior knowledge and most likely yielding a lower quality result.

The reader may protest that what the cruise control actually does is rather simple computation. It takes as input a measurement of the current speed, subtracts it from the desired speed, multiplies by a constant, and adds the result to the current throttle position.^4^ But is this a good description of what the machine does? By analogy, does a human mind take as input a grunt or squeal and produce as output a grunt or squeal? Or does it engage in conversation? Which is a better description? The latter is a description of what the brain, body, and environment accomplish *together*, whereas the former is a description only of what the brain does. The cruise control *system* includes the car, and what it accomplishes is not arithmetic but rather keeping a constant speed.

The cruise control system considered previously could be made more “intelligent” by endowing it with additional feedback. It could check, for example, that when it issues a command to further open the throttle that the car does indeed accelerate. This is a check for fault conditions that might prevent the cruise control system from operating properly. In a way, this check makes the system more “self aware,” aware of its own body and the expected effects that its actions have on that body. Indeed, there is a fledgling subfield of engineering concerned with “self-aware systems,” with a number of workshops worldwide addressing the question of how to design systems that gather and maintain information about their own current state and environment, reason about their behavior, and adapt themselves as necessary. Active interaction with the environment is an essential tool for such systems. The cruise control system has to open the throttle to perform the test to determine whether it is working correctly. Such interaction is a first-person activity, not a third-person observation, and it is a central principle behind embodied robots, which I consider next.

### 6.2. Embodied Robots

Clark and Chalmers (1998) used the term “cognitive extension” for the idea that the mind is not something trapped in the head but rather is spread out into the body and the world around it. Clark's work centers on the processes where the brain tries to predict what the senses will sense and then uses the differences between the predictions and what is sensed to improve the predictions. These feedback loops extend out into the world, encompassing the body and its physical environment so that they become an intrinsic part of thinking. In his words, “certain forms of human cognizing include inextricable tangles of feedback, feed-forward, and feed-around loops: loops that promiscuously crisscross the boundaries of brain, body, and world” (Clark, 2008). If Clark is right, then cognition in machines will not much resemble that in humans until they acquire ways to interact with the world like humans. Some computer programs are already starting to do this, particularly those that control robots.

Robots are, in a sense, embodied computers, but for the most part, they have not been designed in an embodied way. Clark (2008) compares Honda's Asimo robot to humans, observing that Asimo requires about sixteen times as much energy as humans to walk, despite being shorter and lighter. He attributes this to the style of control:

Whereas robots like Asimo walk by means of very precise, and energy-intensive, joint-angle control systems, biological walking agents make maximal use of the mass properties and biomechanical couplings present in the overall musculoskeletal system and walking apparatus itself (Clark, 2008).

Clark points to experiments with so-called passive-dynamic walking (McGeer, 2001). Passive-dynamic robots are able to walk in certain circumstances with *no* energy source except gravity by exploiting the gravitational pull on their own limbs. You can think of these robots as performing controlled falling. McGeer's robots did not include any electronic control systems at all, but subsequent experiments have shown that robots that model their own dynamics in gravity can be much more efficient.

Conventional robotic controllers use a mechanism called a servo, a feedback system that drives a motor to a specified angle, position, or speed. For example, to control a robot arm or leg, first a path-planning algorithm determines the required angles for each joint, and then servos command the motors in each joint to move to the specified angle. The servos typically make little use of any prior knowledge of the physical properties of the arm or leg, their weight and moment of inertia, for example. Instead, they rely on the power of negative feedback to increase the drive current sufficiently to overcome gravity and inertia. It's no wonder these mechanisms are not energy efficient. They are burning energy to compensate for a lack of self-awareness.

Brooks (1992) articulates a vision of “embodied robots” that learn how to manipulate their own limbs rather than having hard-coded, preprogrammed control strategies. Gallese et al. (2020) observe,

Newell and Simon's physical symbol system hypothesis was questioned when the “embodied robots” designed by Rodney Brooks proved able to simulate simple forms of intelligent behavior by externalizing most of cognition onto the physical properties of environments, thus dispensing with abstract symbolic processing” (Brooks, 1991, p. 377).

Brooks' vision was perhaps first demonstrated in real robots by Bongard et al. (2006). Their robot learns to pull itself forward using a gait that it develops by itself. The robot is not even programmed initially to know how many limbs it has nor what their sizes are. It makes random motions initially that are ineffective, much like an infant, but using feedback from its sensors it eventually puts together a model of itself and calibrates that model to the actual limbs that are present. This resembles the learning process in DNNs, where initial decisions are random and feedback is used to improve them. When a leg is damaged, the gait that had worked before will no longer be effective, but since it is continuously learning, it will adapt and develop a new gait suitable for its new configuration. If one of its legs “grows” (someone attaches an extension to it, for example), the robot will again adapt to the new configuration.

Pfeifer and Bongard (2007) assert that the very kinds of thoughts that we humans are capable of are both constrained and enabled by the material properties of our bodies. They argue that the kinds of thoughts we are capable of have their foundation in our embodiment, in our morphology and the interaction between the brain, the body, and its environment. Pfeifer and Bongard argue that fundamental changes in the field of artificial intelligence over the past two decades yield insights into cognition through “understanding by building.” If we understand how to design and build intelligent embodied systems, they reason, we will better understand intelligence in general. Indeed, DNNs are teaching us that intelligence is not necessarily rational.

The classical servo-based robot control systems are simple feedback control loops like those developed by Black (1934). With a servo, the controller plans a path, and the mechanism forces the motion to match that path. Only recently have servos been realized using computers. In embodied robots, a second feedback loop is overlaid on this first one. In this second loop, the robot learns its own morphology and dynamics.

Higher-order cognitive feedback loops also enable humans to recognize flaws in our plans and attempt to improve them. Supporting Clark's argument, in *I Am a Strange Loop*, Hofstadter states:

You make decisions, take actions, affect the world, receive feedback, incorporate it into your self, then the updated “you” makes more decisions, and so forth, round and round (Hofstadter, 2007).

Hofstadter emphasizes that feedback loops create many if not all of our essential cognitive functions. These feedback loops are entirely absent in Turing-Church computation.

### 6.3. Solving Undecidable Problems

Although it is complex, given good enough models, the cruise control and robotics problems are solvable, at least approximately, by a Turing-Church computation. Hence, these arguments do not, by themselves, speak to any *fundamental* limitations of Turing-Church computations.

Interactive systems, however, can sometimes solve problems that are provably unsolvable by Turing-Church computations. Back in the 1990s, a Ph.D. student of mine, Thomas Parks, surprised me by showing how to solve an undecidable problem (Parks, 1995). The problem was to determine whether a particular network of communicating processes built in a particular style due to Kahn and MacQueen (1977) can be executed for an unbounded amount of time using only a bounded amount of memory. Parks proved that the problem is undecidable, meaning that there is no Turing-Church computation that can yield an answer for all possible such networks. He then proceeded to solve the problem with an *interactive* solution. He provided a policy that provably uses bounded memory for any such network that can be executed in bounded memory.^5^ Parks' solution is interactive, in that his scheduling policy makes decisions, watches how the program responds, and makes additional decisions accordingly. Such a strategy does not fit the Turing-Church model.

Strictly speaking, Parks doesn't really solve an undecidable problem, but rather solves a different but related problem. The question he answers is not *whether* a Kahn-MacQueen program can execute in bounded memory, but rather *how* to execute a Kahn-MacQueen program in bounded memory. Computation plays a rather small role in the solution compared to interaction. By analogy, an automotive cruise control is not performing arithmetic; it is keeping the speed of the car constant.

Just as with the cruise control, computation forms a *part* of the interactive machine. Parks' solution performs a Turing-Church computation for each decision. Computers are used this way all the time. A user types something, the machine performs a computation and presents a resulting stimulus to the user, then the user types something more, and the machine performs *another* computation. Each computation is well-modeled by the Turing-Church formalism, but the complete closed-loop system is not.

### 6.4. Reasoning About Causation

Rational decision making frequently involves reasoning about causation. I do not smoke because smoking *causes* cancer. I click on Amazon's website because it *causes* goods to appear at my door. Pearl and Mackenzie (2018) show that it is impossible to draw conclusions about causation in a system by objectively observing the system. One must either *interact* with the system or rely on prior subjective assumptions about causation in the system.

A Turing-Church computation is an objective observer. It does not affect its inputs, as it would if it were an interactive system. To reason about causation, therefore, it can only encode the prior subjective assumptions of its designer. It cannot test those assumptions through interaction. Hence, it is unable to reason about causation.

To understand how interaction helps with reasoning about causation, suppose that we are interested in evaluating whether a particular drug can cause improvements in patients with some disease. In other words, we wish to measure the strength of a hypothesized causal relationship from treatment (whether a treatment is administered) to some measure of health. Suppose that there is risk of some factor that causes a patient to be more or less likely to take the treatment and also affects the patient's health. Such a factor is called a “confounder” in statistics. The confounder could be, for example, gender, age, or genetics. To be specific, suppose that the treatment for some disease is more appealing to women than men, and that women tend to recover more from the disease than men. In that case, gender is a confounder and failing to control for it will invalidate the results of a trial.

In many cases, however, we don't know what confounders might be lurking in the shadows, and there may be confounders that we cannot measure. There might be some unknown genetic effect, for example. We can't control for confounders that we can't measure or that we don't know exist. Is it hopeless, then, to evaluate whether a treatment is effective?

To guard against the risk of hidden confounders, Pearl and Mackenzie (2018) point out, active intervention is effective (when active intervention is possible), underscoring that interaction is more powerful than observation alone. We must somehow force the treatment on some patients and force the lack of treatment on others. Then controlling for the confounding factor is no longer necessary.

Randomized controlled trials (RCTs), are the gold standard for determining causation in medical treatments and many other problems. The way an RCT works is that a pool of patients is selected, and within that pool, a randomly chosen subset is given the drug and the rest are given an identical looking placebo. Ideally, both the patients and the medical personnel are unaware of who is getting the real drug, and the choice is truly random, unaffected in any way by any characteristic of the patients. The system is now interactive because we have forced the value of one of the variables, whether the drug is taken, for each of the patients, and then we observe the results.

RCTs are actually routinely used in software today. It is common at Facebook, for example, when considering a change to the user interface, to randomly select users to whom a variant of the user interface is presented. The reactions of the users, whether they click on an ad, for example, can be measured and compared to a control group, which sees the old user interface. In this way, Facebook software can determine whether some feature of a user interface causes more clicks on ads. This process can be automated, enabling the software to experiment and learn what causes users to click on ads. This is a much more powerful form of reasoning than mere correlation, and it can result in software designing and refining its own user interfaces. The software can even learn to customize the interface for individual users or groups of users. This software is not realizing a Turing-Church computation because it is interactive. The users are an intrinsic part of the system.

It is not always possible or ethical to conduct an RCT. Pearl and Mackenzie (2018) document the decades-long agonizing debate over the question of whether smoking causes cancer. Had it been possible or ethical to randomly select people and make them smoke or not smoke, the debate may have been over much earlier. Instead, we were stuck with tragic observation, watching millions die.

### 6.5. Act to Sense

Two of the three limitations in human rationality identified by Simon (1972), uncertainty about the consequences that would follow from alternative decisions and incomplete information about the set of alternatives, reflect limited information about the environment. Simon zeroed in on the limited ability humans have to *process* information from the environment, but there are also limitations in our ability to *sense*, to gather information from the environment.

Goddfrey-Smith (2016) tells us that sensing is greatly enhanced by feedback. He points out that you need not just *sense-to-act* connections, which even bacteria have, but also *act-to-sense*. To have cognitive function, you have to affect the physical world and sense the changes. Sense-to-act is open loop; you sense, you react. Combine this with act-to-sense, and you close the loop, creating a feedback system.

Goddfrey-Smith (2016) gives a rather nice example of act-to-sense in cephalopods, such as cuttlefish and octopuses, which can change the color of their skin for camouflage and communication. It turns out that cuttlefish are colorblind, having only a single type of photoreceptor molecule. But these molecules are also found in the skin, and by modulating the chromatophores to change the color of the skin, the cephalopod creates a color filter for the light that enters the skin. Dynamically varying the filter reveals the color distribution of the incoming light. They “see” color through their skin *via* a sense-to-act, act-to-sense feedback loop.

Turing-Church computations can only sense-to-act. The formalism does not include any mechanism by which the computation can affect its own inputs.

### 6.6. Efference Copies

Sense-to-act and act-to-sense feedback loops are present in many higher level cognitive functions. Since at least the 1800s, psychologists have studied the phenomenon that the brain can internally synthesize stimulus that would result from sensing some action commanded by the brain. This internal feedback signal is called an “efference copy.” In speech production, for example, while the body is producing sounds that the ears are picking up, at the same time, the brain generates an efference copy, according to this theory, which is fed back into a different part of the brain that calculates what the ears should be hearing, an “expected reafference.” The brain then compensates, adjusting the motor efference to make the speech sound more closely resemble the expectation.

Many psychologists today believe that efference copies help distinguish self-induced from not self-induced sensory stimulus. All animals with sensors have evolved some form of efference copy mechanism because otherwise they would react to their own actions as if those actions were imposed by their environment.

The importance of the efference copy has been understood at some level since the nineteenth century. Grüsser (1995) gives a history, crediting a book by Johann Georg Steinbuch (1770–1818) that illustrated the essential concept with a simple experiment. He noticed that if you hold your hand still and roll an object around in it, say, a spoon, you will not be able to recognize the object from the sensations coming from your hand. But if you actively grasp and manipulate the object, you will quickly recognize it as a spoon. The motor efference, therefore, must play a role in recognition, which implies that the motor efference must be fed back to the sensory system.

Central to this thesis is that our knowledge of the world around us is not solely determined by stimulus that happens to arrive at our sensory organs, but rather is strongly affected by our actions. Without these feedback loops, we would not only suffer limited ability to perform the information processing on our inputs, but we would also have fewer and less meaningful inputs. Turing-Church computations have no efference copies and hence no mechanisms for gathering these more meaningful inputs.

### 6.7. Indiscernable Differences

Lest the reader assume that act-to-sense only makes sensing more efficient, I will now give two rather technical demonstrations that act-to-sense enables making distinctions that are not discernible without feedback. The first of these is the Brock-Ackerman anomaly, a well-known illustration in computer science that observation alone cannot tell the difference between two significantly different systems.

**Figure 1:**
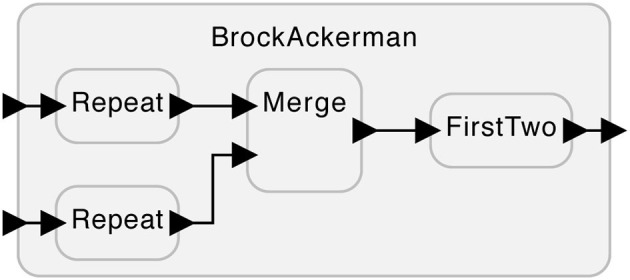
A system with two variants that cannot be distinguished without feedback.

Consider the system shown Figure 1. This system has two inputs at the left that can accept sequences of numbers and one output at the right that produces a sequence of numbers. The subsystems labeled “Repeat” take each input number and repeat it twice on their outputs. For example, if the input to the top Repeat is the sequence (1, 2), the output will be the sequence (1, 1, 2, 2). The subsystem labeled “Merge” arbitrarily interleaves the input sequences it receives on its two inputs. For example, given the two input sequences (1, 2) and (3, 4), it can produce any of (1, 2, 3, 4), (1, 3, 2, 4), (1, 3, 4, 2), (3, 4, 1, 2), (3, 1, 4, 2), or (3, 1, 2, 4). If merge receives nothing on one of its inputs, then it will simply produce whatever it receives on the other input. The subsystem labeled “FirstTwo” simply outputs the first two inputs it receives. For example, given (1, 2, 3, 4), it will produce (1, 2).

Brock and Ackerman (1981) then gave two subtly different realizations of the FirstTwo subsystem:

1. The first realization produces outputs as it receives inputs. That is, as soon as it sees a 1 on its input, it will produce 1 on its output.
2. The second realization waits until there are two inputs available before producing any output. That is, it will not produce any output until both 1 and 2 are available, at which point it will produce the sequence (1, 2).

To an outside observer that can only passively watch the behavior of this system, these two realizations are indistinguishable. The possible output sequences are exactly the same for the same input sequences. For example, if the system is presented with inputs (5) and (6), i.e., two sequences of length one, the possible outputs for either realization are (5, 5), (5, 6), (6, 5), and (6, 6). The choice of realization has no effect on these possibilities.

**Figure 2:**
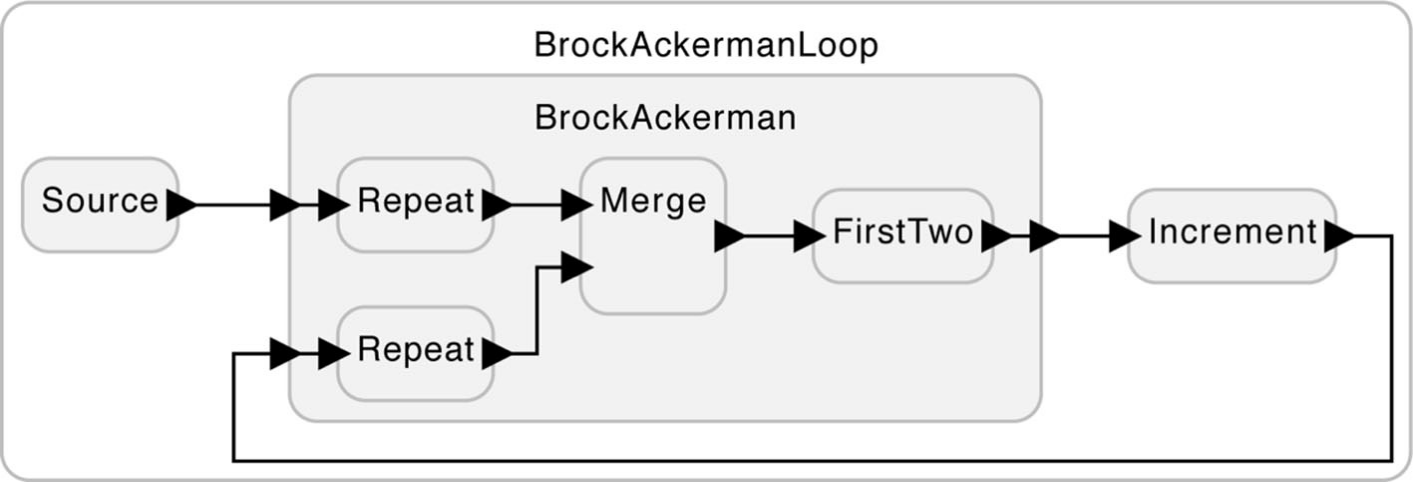
A use of the system in Figure 1 where the two variants of the FirstTwo subsystem yield different behaviors.

Nevertheless, the two realizations yield different behaviors in some circumstances. Consider the system in Figure 2. The subsystem labeled “Increment” simply adds one to each input. For example, given the input sequence (1, 2), it will produce (2, 3). In this usage, it makes a difference which of the two realizations of FirstTwo is used. Suppose that the subsystem labeled “Source” provides on its output the length-one sequence (5). Under realization (1) of FirstTwo, there are two possible outputs from the BrockAckerman subsystem, (5, 5) and (5, 6). But under realization (2), there is only one possible output, (5, 5).

With this example, Brock and Ackerman (1981) proved that two systems that are indistinguishable by a passive observer cannot be substituted one for the other without possibly changing the behavior. They are not equivalent. The feedback of Figure 2 can be thought of as an “embodiment,” where, by interacting with its environment, an otherwise indiscernible difference becomes evident.

Note that the Brock-Ackerman system is not a Turing-Church computation because of the non-deterministic Merge subsystem. The Turing-Church theory admits no such non-determinism. In Chapter 12 of Lee (2020), I show that a passive observer, one that can only see the inputs and outputs of a system, cannot tell the difference between such a non-deterministic system and a deterministic one (a deterministic one *would* be a Turing-Church computation). Only through interaction with the system is it possible to tell the difference. That argument depends on another celebrated result in computer science, Milner's concept of bisimulation.

### 6.8. Milner's Bisimulation

Milner (1980) developed a relation between systems that he called “simulation,” where one system *A* “simulates” another *B* if, given the same inputs, *A* can match the outputs that *B* produces. Park (1980) noticed that there exist systems where *A* simulates *B* and *B* simulates *A*, but where the two systems are not identical. As with the Brock-Ackerman anomaly, the difference between the two systems is indiscernible to a passive observer, but discernible if you can interact with the system. This prompted (Milner, 1989) to revamp his system of logic and develop a stronger form of equivalence that he called “bisimulation.” He then proved that any two systems that are “bisimilar” are indistinguishable not only to any observer, but also to any interactor. Sangiorgi (2009) gives an overview of the historical development of this idea, noting that essentially the same concept of bisimulation had also been developed in the fields of philosophical logic and set theory.

In Chapter 12 of Lee (2020), I give two possible models of tiny universes, the smallest imaginable universes where one entity in the universe is capable of modeling another entity in the same universe. I show two variants of entities in such a tiny universe, one where it is possible that the entity has free will, and one where the entity cannot possibly have free will. I then show that by passive observation alone, it is impossible to tell which entity you are modeling. But if interaction is allowed (using a bisimulation relation), the difference between the two entities can eventually become discernible to any desired degree of certainty. The two entities are not bisimilar. Without detailed knowledge of the inner structure of the entity being modeled, it is not possible to achieve 100% confidence in any conclusion about which entity is being modeled, but through repeated experiments, it is possible to get as close to 100% as you like.

Milner's simulation and bisimulation relations are relations between the possible *states* of two systems. Stretching a bit, one can imagine using these concepts to more deeply understand the relationship between mental states in a cognitive mind and the outside world that those states refer to. Philosophers use the term “intentionality” for such relationships, “the power of minds to be about, to represent, or to stand for, things, properties and states of affairs” outside the mind (Jacob, 2014). Searle (1983) argues that intentionality is central to cognition. Intentionality is about models of the universe that we construct in our brains. Dennett (2013) suggests the less formal term “aboutness” for intentionality. The relationship between mental states and the things that those states are about is essentially a modeling relationship. Milner shows us that such modeling works better when there is dialog, bidirectional interaction, or feedback. It may be that intentionality would likely not arise in a brain that can only observe the world. It must also be able to affect the world.

## 7. Conclusions

In Simon's “bounded rationality,” rationality is the principle that humans make decisions on the basis of step-by-step (algorithmic) reasoning using systematic rules of logic to maximize utility. It becomes natural to equate rationality with Turing-Church computation. However, Turing-Church computation provides no mechanism for *interaction* or *feedback*, where the process provides outputs to its environment that then affect its inputs. The principle of embodied cognition suggests that human decision makers make use of feedback mechanisms for many of our cognitive functions, including rational decision making. Embodied bounded rationality, therefore, suggests that a rational decision maker goes beyond Turing-Church computation, even if the ability to handle computational complexity is limited.

I have given a series of illustrations that show that interaction enables capabilities that are inaccessible to Turing-Church computation, including controlling a system in an uncertain environment, reasoning about causation, solving some undecidable problems, and discerning distinctions between certain kinds of systems. Bounded rationality, therefore, is not the same as a limited capacity to carry out Turing-Church computations because rational processes with feedback are capable of things that Turing-Church computations are not.

Interaction is the core idea in embodied cognition, which posits that a cognitive mind *is* an interaction of a brain with its body and environment. So, while it is true that the human brain has limited Turing-Church computational capability, it also transcends such computation by interacting with its body and environment. Key features of cognition, such as the ability to distinguish self from non-self and the ability to reason about causation, depend on such interaction. Since such interaction is missing from the Turing-Church theory of computation, the “universality” of such computation falls far short of true universality.

Deep neural networks, which have led to a revolution in artificial intelligence, are both interactive and not fundamentally algorithmic. Their ability to mimic some cognitive capabilities far better than prior algorithmic techniques based on symbol manipulation (“good old-fashioned AI”) provides empirical evidence for the power of embodied bounded rationality.

## Author Contributions

The author confirms being the sole contributor of this work and has approved it for publication.

## Conflict of Interest

The author declares that the research was conducted in the absence of any commercial or financial relationships that could be construed as a potential conflict of interest.

## Publisher's Note

All claims expressed in this article are solely those of the authors and do not necessarily represent those of their affiliated organizations, or those of the publisher, the editors and the reviewers. Any product that may be evaluated in this article, or claim that may be made by its manufacturer, is not guaranteed or endorsed by the publisher.

## Footnotes

^1^Copeland (2017) has a nice section on common misunderstandings of the Turing-Church thesis.

^2^For a particularly bad exposition of this hypothesis, full of pseudo science and misinformation, see Virk (2019).

^3^Piccinini (2020) nevertheless defends a “computational theory of cognition” (CTC), though his use of computation is again broader than mine here, and even then, he admits that this theory may not provide a complete explanation.

^4^What I have just described is the simplest form of a negative feedback controller, which is known as a proportional controller. A modern cruise control would more likely realize a PID controller (proportional, integral, derivative), but the computation would only be a slightly more complicated.

^5^This work was later generalized by Geilen and Basten (2003).
